# Data-Driven Efficiency Benchmarking for Risk Mitigation: A Data Envelopment Analysis of U.S. Hospitals, 2018–2024

**DOI:** 10.3390/healthcare14152273

**Published:** 2026-07-25

**Authors:** Diane Dolezel, Suhila Sawesi

**Affiliations:** 1 Health Informatics & Information Management Department, Texas State University, Round Rock, TX 78665, USA; 2 Health and Bioinformatics Program, Department of Information Sciences and Technologies (IST), College of Computing, Grand Valley State University, Grand Rapids, MI 49503, USA; sawesis@gvsu.edu

**Keywords:** hospital efficiency, data-driven healthcare, data envelopment analysis, resource utilization, risk management, health information technology, quality performance, hospital value-based purchasing

## Abstract

**Highlights:**

**What are the main findings?**

- Most U.S. short-term acute care hospitals operated below the DEA efficiency frontier between 2018 and 2024.
- Efficient benchmark hospitals consistently maintained higher IT operating expenditures, while larger hospital size and greater case mix complexity were associated with higher efficiency

**What are the implications of the main findings?**

- DEA benchmarking provides a systems-level perspective for identifying unrealized opportunities to improve care delivery, quality, and patient safety, while informing capacity planning and performance improvement.
- The observed association between health IT spending and efficiency reflects structural patterns rather than a causal relationship.

**Abstract:**

**Objectives**: Hospital efficiency provides a systems-level perspective for identifying unrealized capacity to improve care delivery, quality, and patient safety. This study evaluated relative technical efficiency among U.S. short-term acute care hospitals (2018–2024) and evaluated how structural IT operating investments are converted into clinical service volume and quality-related performance. **Methods**: A longitudinal panel of 8589 hospital-year observations was utilized to estimate technical efficiency with an output-oriented Data Envelopment Analysis (DEA) under variable returns to scale. A secondary Simar–Wilson double-bootstrapped truncated regression (*n* = 2147 complete casesexamined associations with bias-corrected efficiency, clinical quality, case mix, and operational scale. **Results**: Mean DEA efficiency was 0.567, with 7.38% (*n* = 634) operating on the annual efficiency frontier and a mean output expansion potential of 102.49%. Efficient hospitals maintained higher IT operating spending. Stage 2 regression showed that higher Hospital Value-Based Purchasing quality performance was significantly associated with greater inefficiency. Conversely, higher case mix complexity and larger operational scale were associated with higher efficiency. **Conclusions**: Most hospitals operated below the best-practice frontier, indicating gaps in converting resources into service volume and quality. Because core operational drivers were included in the primary DEA model, observed associations show descriptive structural patterns rather than direct cause-and-effect relationships.

## 1. Introduction

United States (U.S.) hospitals operate under sustained financial and operational pressure to deliver high-quality, safe care while containing costs [1]. Workforce shortages, increased reliance on high-cost contract labor, supply-cost inflation, and compressed operating margins, each intensified during and after the COVID-19 pandemic, have renewed interest in how efficiently hospitals convert their resources into care [2,3]. Efficiency in this sense is not a narrow financial construct but a systems-level property: the degree to which a hospital transforms its structural inputs into service volume and quality-related performance. When that transformation falls short of what comparable hospitals achieve with similar resources, the resulting gap represents capacity that is, in principle, recoverable without additional resource inputs.

Data Envelopment Analysis (DEA) is a nonparametric benchmarking method well suited to this question. Rather than assuming a functional form for the production process, DEA constructs an empirical best-practice frontier from observed input–output combinations and measures each unit’s distance from that frontier [4,5,6]. The approach has been applied extensively to hospital performance and is a standard tool in health services efficiency research [7,8,9]. Its appeal for this study is threefold: it accommodates multiple inputs and outputs simultaneously, it identifies peer benchmarks for each hospital, and it expresses inefficiency as an interpretable output gap relative to achievable performance.

Three related bodies of literature bear on hospital performance, efficiency, quality, and patient safety, and health information technology (IT), yet they have largely developed in parallel. Efficiency studies have tended to emphasize financial or service-volume outcomes; patient-safety research has centered on adverse events and clinical quality measures; and health IT research has concentrated on adoption and functionality. Comparatively few studies evaluate the relationship between operational service frontiers and standardized national quality measures, such as the Hospital Value-Based Purchasing (VBP) Total Performance Score, inside a single longitudinal efficiency frontier. Fewer still examine whether IT operating expenditure is associated with measured efficiency in a large national sample spanning the pre- and post-pandemic period [10,11,12].

Health IT is, moreover, a double-edged infrastructure. The same interconnected digital systems that can streamline coordination, reduce process variation, and support clinical decision-making also expand the cybersecurity attack surface and the exposure of sensitive patient data, risks that are themselves patient-safety concerns [13,14]. Framing efficiency as a systems-level indicator of unrealized care delivery and quality-related capacity, therefore, situates this study within the broader question of how digital infrastructure shapes both the capacity to deliver safe care and the vulnerabilities that accompany it.

Against this backdrop, the present study evaluates the relative efficiency of U.S. short-term acute care hospitals from 2018 to 2024 using an output-oriented variable returns to scale DEA model. It also examines whether IT operating expense and other hospital characteristics are associated with variation in efficiency. Because the HVBP Total Performance Score is a composite quality-performance measure rather than a direct measure of adverse events or clinical harm, it is interpreted as a proxy for quality-related performance. Because the COVID-19 pandemic affected hospital operations and led to HVBP program revisions, data exclusions, and rescoring, post-2020 efficiency patterns are interpreted cautiously. The remainder of the paper develops the conceptual framework, specifies the model and data, reports the efficiency estimates, and discusses the implications for resource optimization and clinical risk mitigation.

### 1.1. Background and Gap in the Literature

Prior research has examined hospital efficiency, patient safety, quality performance, and health IT adoption as largely separate areas of study [7,8,9]. Efficiency research has often focused on financial or service-volume outcomes, while patient safety research has emphasized adverse events and clinical quality measures. Similarly, health IT studies have concentrated on adoption and functionality, with limited attention to the relationship between IT operating expense and overall hospital efficiency [10].

Furthermore, limited empirical evidence evaluates hospital efficiency as a systems-level indicator of unrealized quality and patient safety performance. Few studies have integrated operational outputs with standardized national quality performance measures, such as the Hospital Value-Based Purchasing Total Performance Scores, within a single longitudinal efficiency framework. In addition, the association between IT operating expenditures and hospital efficiency is not well understood across the pre- and post-COVID-19 pandemic period. This study addresses that gap by integrating operational outputs, a national quality performance measure, and IT operating expense within a longitudinal DEA framework.

### 1.2. Conceptual Framework

This study is guided by a systems-based perspective informed by general systems theory and the Donabedian structure–process–outcome model of healthcare quality [15,16]. Hospitals are conceptualized as complex systems in which structural inputs (e.g., capacity, labor, and health information technology (IT) resources) are transformed through care processes into outputs (e.g., service volume and quality performance). Within this framework, Data Envelopment Analysis (DEA) is applied as a data-driven benchmarking approach to estimate the efficiency of this transformation and identify best-practice frontiers [4,5,6].

Efficiency is interpreted as a systems-level indicator reflecting how effectively hospitals utilize available resources to deliver care. Inefficiency represents an output gap, defined as the difference between observed and achievable performance, given existing inputs. From a patient safety perspective, this output gap may be interpreted, with appropriate caution, as a potential indicator of latent clinical risk, that is, unrealized capacity to deliver timely, effective, and high-quality care, rather than as a direct measure of harm.

Health IT is positioned as an enabling infrastructure that may improve coordination, reduce variability in care processes, and support clinical decision-making [10,11,12]. Accordingly, IT investment may reflect investment in a data-driven infrastructure that supports resource optimization and quality-related clinical risk management. The same infrastructure, however, expands cybersecurity and data-privacy exposure that bears directly on patient safety [13,14]. This study evaluates these relationships empirically within a national sample of U.S. hospitals.

### 1.3. Study Purpose

The purpose of this study is to evaluate hospital efficiency as a systems-level indicator of care delivery capacity, quality performance, and institutional sustainability among U.S. short-term acute care hospitals from 2018 to 2024. Using a two-stage design, the study first estimates relative efficiency with an output-oriented variable returns to scale (VRS) Data Envelopment Analysis model built on contemporary, annual frontiers stratified by geographic location and ownership type.

The secondary objective is to evaluate how these underlying technical efficiency scores relate to quality benchmarks, specifically the Hospital Value-Based Purchasing (HVBP) Total Performance Score, along with operational controls, including patient acuity (Case Mix Index). By identifying hospitals operating below achievable performance benchmarks, this study aims to identify ways hospitals can improve the quality and efficiency of care without needing to spend more money or increase resource inputs.

### 1.4. Research Questions and Hypotheses

The primary research question is: To what extent is a hospital’s technical efficiency associated with its performance outcomes under value-based reimbursement frameworks, after accounting for temporal shifts, geographic location, and institutional characteristics?

Based on this research question, the hypotheses are:

**H1.**  *Technical efficiency scores will be substantially below the separate annual efficiency frontier for most hospital-year observations, indicating significant unrealized quality and service capacity within the hospital*.

**H2.**  *Higher hospital quality performance will be positively associated with bias-corrected operational efficiency scores, indicating that clinical quality and operational efficiency are complementary rather than competing institutional goals*.

**H3.**  *Higher patient complexity (Case Mix Index) and larger operational scale (outpatient visits and surgical volumes) will be negatively associated with operational inefficiency distance scores in Stage 2 regression*.

The findings contribute to the literature by demonstrating a rigorous two-stage, Simar–Wilson double-bootstrap approach that separates the measurement of technical efficiency from changes in regulatory scoring systems, disruptions caused by the COVID-19 pandemic, and potential statistical bias. This provides more reliable estimates that reflect true operational performance and adds methodological novelty to the paper.

## 2. Materials and Methods

### 2.1. Study Design

This retrospective longitudinal study evaluated a longitudinal panel of hospital-year observations for operational performance from 2018 to 2024 using a two-stage design. In the first stage, a nonparametric Data Envelopment Analysis (DEA) was applied to calculate technical efficiency scores across hospital decision-making units (DMUs), which is a commonly used method for comparing resource efficiency [4,5,6]. To control for unexpected events and hospital differences, we estimated separate annual DEA frontiers. To ensure hospitals were benchmarked only against direct operational peers, these frontiers were stratified by unique peer groups defined by the intersection of geographic setting (rural vs. urban) and ownership status (Nonprofit, Proprietary, Governmental, and Other).

All first-stage models were analyzed under an output-oriented DEA framework utilizing the variable returns to scale (VRS) assumption. This framework evaluates a hospital’s capability to maximize service volumes given its current resources, and the VRS assumption guarantees that hospitals are benchmarked exclusively against peer facilities operating within a similar scale of operation. This prevents smaller facilities from being compared to large medical centers. In the second stage, a truncated Simar–Wilson regression model was utilized to examine the institutional and environmental factors associated with these efficiency scores.

To improve intuitive economic interpretability in Stage 1 reporting, output-oriented DEA efficiency scores were expressed on a 0 to 1 scale. On this scale, a score of 1.00 indicates a fully efficient hospital operating on the empirical frontier, while values below 1.00 indicate lower operational efficiency. For example, an efficiency score of 0.70 implies that the hospital achieved 70% of its benchmark output potential given its observed structural inputs, indicating a 30% capacity shortfall. For second-stage econometric modeling under the rDEA framework, scores were specified as Shephard distance functions, where values equal to 1.00 denote efficiency and values exceeding 1.00 measure distance from the frontier (inefficiency).

### 2.2. Data and Sampling

Data were obtained from the Definitive Healthcare database for the 2018–2024 study period. Definitive Healthcare provides comprehensive information on U.S. healthcare organizations, including hospital characteristics, financial performance, and operational metrics [17]. The database integrates information from multiple sources, including the American Hospital Association Annual Survey for hospital profile data, Medicare Cost Reports for financial data, the Hospital Value-Based Purchasing Program for quality data, and Hospital Compare for publicly reported quality measures.

The initial dataset included 23,721 hospital-year observations from short-term acute care hospitals. Hospitals were eligible for inclusion if they were short-term acute care hospitals, were not identified as closed, and had complete, nonnegative data for all input and output variables used in the DEA model.

During data cleaning, a total of 15,132 hospital-year observations were excluded because they had missing values for one or more DEA input or output variables or were identified as closed. No observations were excluded because of negative values. Because the DEA requires complete, nonnegative data, observations with missing input or output values were excluded using a complete-case approach. Because complete-case analysis was used for the primary sample, the analytic sample may overrepresent hospitals with more complete reporting if missingness was associated with hospital size, resources, or reporting capacity.

After applying these exclusion criteria, the final analytic sample included 8589 complete, nonnegative hospital-year observations from 1227 unique hospitals. A secondary complete-case subsample of *n* = 2147 hospital-year observations with complete longitudinal Hospital Value-Based Purchasing (HVBP) Total Performance Scores was evaluated in the Stage 2 truncated regression modeling.

### 2.3. Stage 1 DEA Input and Output Variables

The first-stage DEA model included the input variables: staffed inpatient beds, annual contract labor expense, and IT operating expense. The output variables were: inpatient_revenue and outpatient revenue. To evaluate technical efficiency, we utilized an output-oriented model under variable returns to scale (VRS) assumptions to account for varying hospital sizes. Staffed inpatient beds measured hospital service capacity, contract labor expense measured purchased labor resources, and IT operating expense reflected the annual spending for ongoing IT support. These inputs have been associated with operational performance and quality improvement [10,11,12]. All inputs were specified so that lower input use indicated greater efficiency.

The financial variables captured the institutional output and service delivery across both overnight and ambulatory settings. The Hospital Value-Based Purchasing (HVBP) Total Performance Score was intentionally excluded from these first-stage DEA frontier calculations to prevent the quality framework from influencing the baseline technical efficiency estimates.

### 2.4. Stage 2 Simar–Wilson Regression Covariates

Stage 2 employed a Simar–Wilson truncated regression model in which the bias-corrected DEA efficiency scores were regressed on quality, case mix, staffing, and volume covariates. To maintain computational tractability under the intensive Simar–Wilson double-bootstrap procedure (*N* = 2147, L_1_ = 100, L_2_ = 1000 replications, requiring 100,000 linear programming optimizations per run), a reproducible 25% random subsample (*N* = 2147) was drawn from the full analytic sample (*N* = 8589).

The explanatory variables included HVBP Total Performance Score, Case Mix Index, Readmission Rate, Annual Outpatient Visits, and Total Surgical Procedures. Outpatient visits and total surgical procedures were included to capture operational scale and clinical throughput beyond financial output measures used in the DEA model.

The regression model formula is:

$${\mathrm{Distance}}_{i}=\alpha +\beta_{1}{\mathrm{HVBP}}_{i}+\beta_{2}{\mathrm{CMI}}_{i}+\beta_{3}{\mathrm{Readmission}}_{i}+\beta_{4}{\mathrm{OutpatientVisits}}_{i}+\beta_{5}{\mathrm{Surgeries}}_{i}+\varepsilon_{i}$$

where Distance*_i_* is the bias-corrected DEA Shephard distance score (inefficiency), HVBP is the Hospital Value-Based Purchasing Total Performance Score, and CMI is the Case Mix Index.

Because IT operating expense was included only as a first-stage DEA input and was not entered as a second-stage explanatory variable, the analysis avoided potential circularity in estimating its relationship with operational efficiency (Table 1).

### 2.5. Data Preparation and Analysis

DEA requires nonnegative input and output values. No negative values were present in the analytic sample. Contract labor expense and IT operating expense were scaled to millions of USD to improve interpretation. All analyses were conducted in R version 4.6.0.

To implement the stratified annual frontier approach, we iterated through each unique peer group and study year, estimating individual DEA models for each subset using the Benchmarking package. First-stage technical efficiency frontiers were generated using the dea() function in the Benchmarking package, while second-stage estimation was conducted using the dea.env.robust() function within the rDEA package. Descriptive statistics were calculated for all DEA variables. Pairwise correlations among variables were examined to assess whether the selected variables captured distinct dimensions of hospital resources and clinical service volume.

In the second stage, we examined the relationship between baseline operational efficiency and quality performance. To avoid endogeneity, primary service outputs and IT operating expenses used to calculate the DEA efficiency scores were not included in the second-stage regression.

Furthermore, because standard ordinary least squares linear regressions are inappropriate for bounded DEA scores, we utilized the Simar and Wilson (2007) robust double-bootstrap procedure [18]. Consequently, the second-stage model regressed the bias-corrected efficiency scores on the HVBP Total Performance Score alongside our designated institutional and environmental control covariates. To ensure the stability and reliability of our estimates, we employed L_1_ = 100 loop repetitions to estimate the bias-corrected efficiency scores and L_2_ = 1000 loop repetitions for the truncated regression bootstrap inference.

## 3. Results

This section presents descriptive characteristics of the sample, temporal trends in DEA inputs and outputs, DEA efficiency estimates, and secondary regression results examining associations between hospital characteristics and efficiency scores.

### 3.1. Descriptive Statistics of DEA Variables

Table 2 presents descriptive statistics for the DEA input and output variables, alongside the hospital quality benchmark score. Hospitals varied considerably in capacity, financial resource use, revenue generation, and quality performance. The mean IT operating expense was 14.18 million USD (median = 7.50 million USD), while the mean number of staffed beds was 245.37 (median = 178.00), with physical capacity ranging widely from 20 to 1893 beds. These substantial spreads suggest that a highly resourced subset of hospitals maintained significantly larger capital and technology infrastructures relative to the rest of the cohort.

Financial production and service value also varied extensively across the hospital-year observations. Outpatient revenue averaged 917.41 million USD (median = 581.02 million USD), spanning from a minimum of 15.45 million USD to a maximum of over 23,371.12 million USD. Similarly, inpatient revenue averaged 903.63 million USD (median = 457.66 million USD), ranging from 10.34 million USD up to 21,743.92 million USD. Reflecting clinical delivery performance, the Hospital Value-Based Purchasing (HVBP) score demonstrated a mean baseline of 32.66 (median = 31.96), with individual facility-year scores extending from a low of 2.50 to a high of 80.83. Overall, these findings indicate substantial variation in hospital size, resource use, service volume, and quality performance, strongly supporting the use of a variable returns to scale (VRS) DEA modeling framework, which allows for robust efficiency comparisons among hospitals operating at completely different scales.

The Pearson correlation analysis indicated that all pairwise correlations between the Stage 2 environmental covariates fell safely below the commonly cited econometric threshold of 0.70 for severe multicollinearity. Stage 1 variables that had strong positive associations were Staffed Beds and IT Operating Expense (r = 0.80), and IT Operating Expense and Outpatient Revenue (r = 0.88), followed by Inpatient and Outpatient Revenues (r = 0.87), but these are below the commonly cited severe multicollinearity threshold of 0.90 (Table 2).

### 3.2. Trends in Inputs and Outputs

Table 3 summarizes trends in hospital inputs and outputs from 2018 to 2024. The average number of staffed beds increased moderately, from 241.58 in 2018 to 248.11 in 2024. In contrast, IT operating expense increased more substantially, from 11.58 to 17.34 million USD across the study years.

Inpatient revenue grew progressively over the study period from baseline averages of 732.12 million USD to 1117.93 million USD. Outpatient revenue experienced even more aggressive growth over the same period, growing from 705.65 million USD to 1210.05 million USD. Additionally, outpatient revenue exceeded inpatient revenue starting in 2022 (974.12 million USD versus 956.98 million USD). While the Case Mix Index slightly increased from 1.656 to 1.752, the Readmission Rate slightly decreased from 15.4% to 14.7%; these measures are reported to three decimal places to capture subtle annual variations. In contrast, HVBP quality scores declined after 2020, falling from 36.97 in 2020 to 21.61 in 2024.

### 3.3. DEA Model Summary (H1)

Table 4 summarizes the output-oriented DEA VRS model results. The output-oriented VRS model identified 634 efficient hospital-year observations, representing 7.38% of the sample. The mean efficiency score was 0.567 (median = 0.526). These results indicate that most hospital-year observations operated substantially below the estimated best-practice frontier. The mean DEA output expansion factor was 2.025, corresponding to a mean expansion potential of 102.49%. The median DEA output expansion factor was 1.902, corresponding to a median output expansion potential of 90.18%. These estimates indicate substantial output gaps under this DEA model, while holding inputs constant.

**Figure 1:**
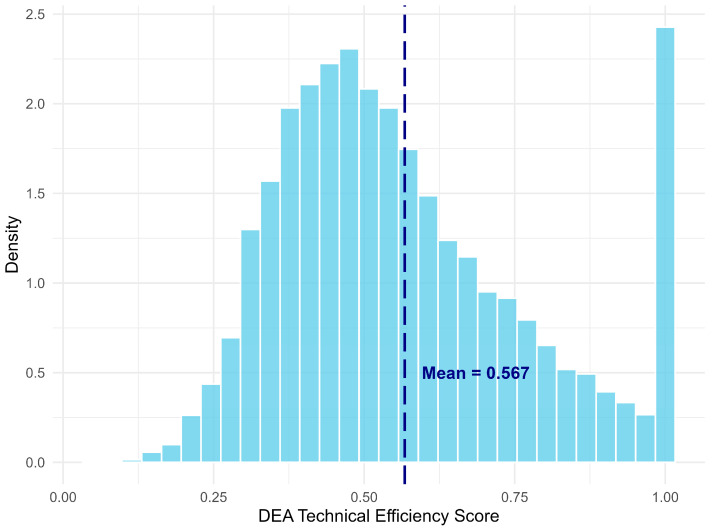
Distribution of DEA technical efficiency scores across hospital-year observations. Scores closer to 1.00 indicate greater relative efficiency.

Figure 1 displays the distribution of the baseline Stage 1 DEA technical scores with a line for the mean efficiency score of 0.567. Most scores are concentrated between 0.25 and 0.75, with a median of 0.526, indicating that few hospital-year observations approach the efficiency frontier. Moreover, the isolated tall bar at 1.00 indicates that only a small proportion of hospital-year observations were fully efficient. Overall, this distribution shows most hospitals operate well below the annual efficient frontier, providing strong evidence of widespread operational inefficiency and unrealized capacity across the hospital sample.

### 3.4. DEA Efficiency by Year

Table 5 presents the distribution of DEA efficiency scores and mean output expansion by year from 2018 to 2024. Mean efficiency scores were highest between 2018 and 2019 (0.577), with median efficiency scores reaching 0.540 in 2018 and 0.535 in 2019. There were approximately 7.3% to 8.2% of facilities on the annual efficient frontier.

Beginning in 2020–2021, efficiency scores declined. The median efficiency score fell from 0.540 in 2018 to a study low of 0.517 in 2021, and the mean efficiency score dropped from 0.577 in 2018 to 0.558 in 2021. The interquartile range also shifted downward over time, as denoted by the decrease in the 25th percentile, dropping from 0.423 in 2018 to 0.399 in 2024, reflecting efficiency reductions for all facilities.

The annual proportion of fully efficient hospitals decreased from a high of 8.15% in 2018 to a low of 6.76% in 2021. Concurrently, mean output expansion increased from approximately 96.29% in 2018 to a high of 107.48% in 2024. Overall, these findings indicate a sustained decline in hospital efficiency from 2019 to 2020, with fewer hospitals operating near the frontier and larger output gaps.

### 3.5. Descriptive Profile of Efficient and Inefficient Hospital-Year Observations (H1 and H3)

Table 6 compares the average input and output characteristics of efficient and inefficient hospital-year observations. On average, efficient hospitals were larger, with a higher mean number of staffed beds when compared to inefficient hospitals (272.70 vs. 243.20 beds, relative to the overall sample mean of 245.37 beds). Resource use showed considerable variation between the groups, especially for IT operating expenses. Efficient hospital-year observations reported higher mean IT operating expenses than inefficient hospital-year observations (17.77 vs. 13.89 million USD), with an absolute mean difference of 3.88 million USD.

Efficient hospitals had substantially higher mean inpatient revenue (1880.16 million USD vs. 825.80 million USD) and outpatient revenue (1678.97 vs. 856.72 million USD), with absolute mean differences of 1054.36 million USD and 822.25 million USD, respectively. Overall, efficient hospital-year observations generated significantly higher revenues relative to their underlying scale and IT resource inputs. Because these variables were used to calculate the Stage 1 DEA efficiency scores, these comparisons should be interpreted as descriptive profiles rather than as evidence of independent predictors of efficiency. Detailed hospital-level listings of the ten most and least efficient hospitals, with names replaced with pseudonyms, are provided in Supplementary Tables S1 and S2.

These descriptive patterns align with H1 by showing that most hospital-year observations operated below the efficiency frontier. Additionally, efficient hospitals had higher mean health IT spending and larger physical capacity measured by staffed beds. However, because these univariate comparisons do not control for confounding factors, H2 (quality performance) and H3 (patient complexity and operational volume scale) are evaluated in the Stage 2 multivariate truncated regression model.

### 3.6. Stage 2 Truncated Regression (H2 and H3)

Table 7 presents the results of the Stage 2 Simar–Wilson output-oriented bootstrapped truncated regression. The Stage 1 DEA efficiency scores were initially calculated across the complete baseline production space (*N* = 8589), stratified by annual peer-group frontiers to establish benchmarks. For the Stage 2 evaluation, a seed-reproducible 25% random sample (*N* = 2147) was drawn. Per the Shephard distance function specification in *rDEA*, the dependent variable is the reciprocal of technical efficiency (1.0 = text{fully efficient}; values > 1.0 indicate operational inefficiency). Consequently, positive coefficients denote an increase in inefficiency, whereas negative coefficients denote an increase in operational efficiency.

Because this double-bootstrapped procedure does not produce traditional *p*-values, statistical significance was assessed using bias-corrected 95% confidence intervals generated across 2000 bootstrap iterations. An effect was considered statistically significant at the 5% level if its 95% confidence interval excluded zero. All reported explanatory variables and control metrics met this criterion, indicating statistically significant associations with operational efficiency distance.

Hypothesis 2, which proposed that higher hospital quality was positively associated with operational efficiency, was not supported. The coefficient for the HVBP Total Performance Score is positive and statistically significant (beta = 0.0115, 95% CI [0.0024, 0.0209]), indicating that higher administrative quality scores are associated with greater operational distance from the efficient frontier (i.e., lower technical efficiency).

Hypothesis 3, which proposed that larger operational service scale and patient volume decrease operational inefficiency, was fully supported. Higher physical service volume significantly increases operational efficiency for both outpatient visits (beta = −0.0000007, 95% CI [−0.0000013, −0.0000000]) and total surgeries (beta = −0.0000278, 95% CI [−0.0000458, −0.0000078]), as indicated by their negative distance coefficients. All Stage 1 resource inputs, including IT operating expense, staffed beds, and contract labor expense, were completely excluded from Stage 2 to eliminate potential endogeneity and mechanical circularity.

Among the control variables, the Case Mix Index (CMI) displays a strong negative association with inefficiency distance (beta = −1.5664, 95% CI [−2.0265, −1.1097]), indicating that treating higher-complexity patient populations significantly increases output efficiency relative to inputs. Conversely, the Readmission Rate demonstrates a strong positive relationship with distance scores (beta = 28.5073, 95% CI [16.8267, 40.1577]), confirming that higher clinical Readmission Rates significantly reduce operational efficiency.

## 4. Discussion

The study evaluated hospital efficiency as a systems-level indicator of unrealized care delivery, quality performance, and patient safety potential among U.S. short-term acute care hospitals from 2018 to 2024, utilizing an output-oriented Data Envelopment Analysis model.

### 4.1. Principal Findings

Overall, mean hospital efficiency scores remained substantially below the empirical frontier, demonstrating significant unrealized quality and service capacity across peer hospitals. In Stage 2 truncated regression analysis, higher healthcare quality performance was significantly associated with greater operational inefficiency distance. Conversely, larger operational scale, measured through outpatient and surgical service volumes, demonstrated a significant positive relationship with technical efficiency. Furthermore, higher patient complexity (Case Mix Index) was strongly associated with increased output efficiency, whereas elevated Readmission Rates significantly worsened operational efficiency scores. All baseline resource inputs were completely excluded from the second stage to eliminate endogeneity and ensure the methodological integrity of the two-stage framework.

### 4.2. Interpretation by Hypothesis

Hypothesis 1 proposed that hospital efficiency scores would be substantially below the efficiency frontier for most hospital-year observations. This was fully supported by our Stage 1 DEA results. Most hospital efficiency scores were concentrated at lower efficiency levels, with only a small annual fraction (less than 8.2%) defining the annual frontier, and over 50% operating well below their benchmarked potential output. As an illustration, inefficient hospitals exhibited a mean annual output expansion potential exceeding 100%, peaking at 107.48% in 2024.

These large efficiency gaps reflect operational and economic challenges faced by hospitals in our study years. First, financial data reflects a massive shift toward outpatient care; by 2022, outpatient revenue exceeded inpatient revenue. Second, hospital quality scores dropped notably after 2020. Thus, it is likely the decline reflects where hospitals were challenged to reimagine their care delivery models while simultaneously battling post-pandemic quality drops. While using a balanced panel ensures that the same facilities are tracked over time, Stage 1 DEA models benchmark all facilities against large, highly efficient medical centers, which could cause smaller or specialized hospitals to appear systematically less efficient [8,9]. Stage 2 addresses this frontier estimation bias with the Simar–Wilson double-bootstrap procedure.

Hypothesis 2 proposed that higher clinical quality care would be associated with higher operational efficiency. The Stage 2 truncated regression did not support this prediction. Instead, higher quality performance scores were significantly associated with greater operational distance from the efficient frontier, indicating greater operational inefficiency. This finding points to an ongoing operational trade-off in hospital care, where delivering high-quality, guideline-based care requires additional clinical staffing, documentation overhead, and administrative coordination that can reduce short-term technical efficiency.

Hypothesis 3 proposed that larger operational service scale and higher patient complexity would decrease operational inefficiency. The Stage 2 findings supported this hypothesis. As predicted, higher physical outpatient visit volume and total surgical procedures were significantly associated with lower distance scores, reflecting greater operational efficiency through scale economies. Furthermore, higher patient clinical complexity significantly enhanced efficiency. To ensure methodological rigor and eliminate mechanical circularity, all Stage 1 resource inputs, including contract labor expense, IT operating costs, and staffed beds, were completely excluded from the Stage 2 regression model.

### 4.3. Practical Implication

For hospital leaders and healthcare policymakers, these findings suggest placing less emphasis on increasing spending and greater emphasis on improving the use of existing resources. Because many acute care hospitals in the study demonstrated unrealized quality and service capacity relative to peer institutions with comparable inputs, there is considerable opportunity to optimize operational efficiency. Health system executives can use DEA benchmarking to identify top-performing peer hospitals and adopt operational practices that more effectively convert available resources into high-quality patient care.

Although high-performing hospitals invested more heavily in health information technology to support expanded service capacity, these investments should be accompanied by strong cybersecurity, risk mitigation, and governance frameworks. Maintaining system resilience and protecting patient data are essential, as cyber disruptions can jeopardize patient safety and undermine operational efficiency. Finally, because expanding physical service throughput, such as outpatient visits and surgical procedures, and managing case complexity were shown to significantly improve efficiency, health system executives should focus on optimizing operational scale, workflow productivity, and core workforce retention rather than short-term resource expansion to safeguard long-term financial sustainability.

### 4.4. Limitations

Several limitations of this study should be noted. First, the analysis relied on a strictly balanced panel sample tracking *N* = 1227 unique acute care hospitals across all seven study years (*N* = 8589). While this balanced design prevents sample composition bias across time, hospitals excluded due to missing data or incomplete reporting across any year may overrepresent better-resourced facilities. Second, the HVBP Total Performance Score is an administratively derived proxy for quality that does not capture all adverse events or long-term mortality rates. Third, to prevent mechanical circularity and endogeneity, all Stage 1 resource inputs. including IT operating expense, staffed beds, and contract labor expense, were completely excluded from the Stage 2 regression model. Moreover, health IT operating expenses were strongly correlated with baseline hospital scale, indicating that IT investment closely tracks overall institutional capacity. Finally, all financial variables were derived from the Definitive Healthcare database and are dependent on the accuracy of the definitions in that source, and should be interpreted accordingly.

## 5. Conclusions

This study evaluated technical efficiency across U.S. short-term acute care hospitals from 2018 to 2024 using a strictly balanced panel. By constructing independent annual cross-sectional frontiers and employing a two-stage Simar–Wilson double-bootstrap design, the framework isolates operational throughput from regulatory measurement shifts. The findings reveal substantial unrealized quality and service capacity across peer institutions. While higher physical service volumes (outpatient visits and surgical procedures) and patient case complexity significantly drive operational efficiency, higher quality performance scores reflect a structural trade-off with short-term technical efficiency. Ultimately, double-bootstrapped DEA offers healthcare leaders a robust benchmarking tool to optimize resource conversion, scale service throughput, and strengthen data-driven care delivery.

## Figures and Tables

**Table 1:** Stage 1 DEA Input and Output Variables and Stage 2 Simar–Wilson Truncated Regression Covariates.

Stage	Variable	Role	Rationale
Stage 1	Staffed inpatient beds	Input	Hospital service capacity
	Contract labor expense	Input	Purchased labor resources
	IT operating expense	Input	Health IT investment and support
	Inpatient revenue	Output	Inpatient service production
	Outpatient revenue	Output	Outpatient service production
Stage 2	HVBP Total Performance Score	Covariate	Clinical quality performance
	Case Mix Index	Covariate	Patient complexity
	Readmission rate	Covariate	Care quality outcome
	Annual outpatient visits	Covariate	Operational scale and throughput
	Total surgical procedures	Covariate	Clinical service volume

**Table 2:** Descriptive Statistics for DEA Input and Output Variables (*N* = 8589).

Variable	Mean	SD	Min	p25	Median	p75	Max
Staffed Beds	245.37	218.44	20	98	178	323	1893
IT Operating Expense (Million USD)	14.18	20.56	0.55	3.77	7.5	15.53	277.64
Inpatient Revenue (Million USD)	903.63	1352.87	10.34	162.11	457.66	1114.05	21,743.92
Outpatient Revenue (Million USD)	917.41	1227.99	15.45	306.85	581.02	1061.47	23,371.12
HVBP	32.66	11.52	2.50	24.58	31.96	39.5	80.83

**Table 3:** Trends in Hospital Inputs and Outputs, 2018 to 2024 (*N* = 8589).

Year	Staffed Beds	IT Operating Exp (Million USD)	Inpatient Revenue (Million USD)	Outpatient Revenue (Million USD)	Case Mix Index (CMI)	Readmission Rate (%)	HVBP
2018	241.584	11.575	732.119	705.648	1.656	0.154	35.898
2019	242.796	12.260	784.289	780.266	1.672	0.153	36.762
2020	244.569	12.991	807.223	784.933	1.761	0.156	36.968
2021	246.889	13.876	905.124	877.854	1.766	0.155	32.460
2022	246.751	15.138	956.983	974.119	1.778	0.150	32.460
2023	246.911	16.081	1021.707	1089.031	1.744	0.145	32.460
2024	248.112	17.337	1117.933	1210.054	1.752	0.147	21.605

Note: Readmission Rates are entered as proportions (0.000–1.000) in modeling and reported to three decimal places to preserve subtle annual variations that would otherwise be obscured by rounding.

**Table 4:** Summary of Output-Oriented VRS DEA Efficiency Results (*N* = 8589).

Measures	Value
Total hospital-year observations	8589
Efficient hospital-year observations	634
Inefficient hospital-year observations	7955
Percent efficient (%)	7.38
Mean DEA expansion factor	2.025
Median DEA expansion factor	1.902
Minimum DEA expansion factor	1
25th percentile DEA expansion factor	1.442
75th percentile DEA expansion factor	2.437
Maximum DEA expansion factor	20.598
Mean efficiency score	0.567
Median efficiency score	0.526
Mean output expansion potential (%)	102.49
Median output expansion potential (%)	90.18

Note. Efficiency scores are calculated as the inverse of the DEA output expansion factor; output expansion potential (%) is computed as (expansion factor − 1) × 100.

**Table 5:** DEA Efficiency Scores and Output Expansion by Year (*N* = 8589).

Year	Total *N*	Efficient *N*	Efficient (%)	Mean	Median	25th Percentile	75th Percentile	Mean Output Expansion
2018	1227	100	8.15	0.577	0.540	0.423	0.687	96.29
2019	1227	90	7.33	0.577	0.535	0.425	0.702	96.77
2020	1227	91	7.42	0.568	0.525	0.409	0.695	102.29
2021	1227	83	6.76	0.558	0.517	0.41	0.685	105.69
2022	1227	87	7.09	0.567	0.522	0.41	0.697	102.60
2023	1227	95	7.74	0.564	0.521	0.40	0.687	106.28
2024	1227	88	7.17	0.561	0.518	0.399	0.695	107.48

Note: Efficient *N* and (%) show facilities defining the annual frontier, where lower efficiency scores indicate greater distance from the frontier. Mean Output Expansion (%) is the average percentage increase in outputs required for inefficient hospitals to reach the frontier, holding inputs constant.

**Table 6:** Input and Output Characteristics by Efficiency Group (Group Means) (*N* = 8589).

Variable	Efficient	Inefficient	Absolute Difference
Staffed beds	272.70	243.20	29.5
IT operating expense (Million USD)	17.77	13.89	3.88
Inpatient revenue (Million USD)	1880.16	825.80	1054.36
Outpatient revenue (Million USD)	1678.97	856.72	822.25

Note. Values represent group means for efficient and inefficient hospital-year observations. These differences may reflect skewed distributions influenced by a small number of high-value observations.

**Table 7:** Simar–Wilson Truncated Regression Results (Stage 2) (n=2147).

Variable	Coefficient	95% Lower CI	95% Upper CI
(Intercept)	1.67	−0.3045	3.6495
vbp_score	0.0115	0.0024	0.0209
case_mix_index	−1.5664	−2.0265	−1.1097
readmission_rate	28.5073	16.8267	40.1577
outpatient_visits	−0.0000007	−0.0000013	−0.0000000
total_surgeries	−0.0000278	−0.0000458	−0.0000078

Note: The dependent variable is the Stage 1 bias-corrected DEA Shephard distance score (higher values indicate lower operational efficiency). Statistical significance at *p* < 0.05 is indicated by confidence intervals that do not include zero.

## Data Availability

Datasets were accessed with the assistance of the Definitive Healthcare website (found here: https:www.defhc.com, accessed on 18 May 2025). This is a subscription-based resource. As such, data are proprietary and cannot be publicly reposted, redistributed, or shared.

## Supplementary Materials

The following supporting information can be downloaded at: https://www.mdpi.com/article/10.3390/healthcare14152273/s1, Table S1: Top 10 Most Efficient Hospital-Year Observations; Table S2: Top 10 Least Efficient Hospital-Year Observations.

## Abbreviations

The following abbreviations are used in this manuscript:

AHA	American Hospital Association
CMS	Centers for Medicare & Medicaid Services
COVID-19	Coronavirus Disease 2019
DEA	Data Envelopment Analysis
DMU	Decision-Making Unit
HVBP	Hospital Value-Based Purchasing
IT	Information Technology
U.S.	United States
VBP	Value-Based Purchasing
VRS	Variable Returns to Scale
