# Supplementary material for: Data-Driven Efficiency Benchmarking for Risk Mitigation: A Data Envelopment Analysis of U.S. Hospitals, 2018–2024
Source: Healthcare (Basel). 2026 Jul 25;14(15):2273. doi: 10.3390/healthcare14152273 (PMC13464019; doi:10.3390/healthcare14152273)
Supplement: Supplementary file 1 [file healthcare-14-02273-s001.zip › healthcare-4432190-supplementary.pdf]

Data-Driven Efficiency Benchmarking for Risk Mitigation: A Data Envelopment Analysis of U.S. Hospitals, 2018–2024

Table S1. Top 10 Most Efficient Hospital-Year Observations

Variable	HospE1	HospE2	HospE3	HospE4	HospE5	HospE6	HospE7	HospE6	HospE9	HospE10
**DEA Efficiency Score**	1.00	1.00	1.00	1.00	1.00	1.00	1.00	1.00	1.00	1.00
**Rural / Urban Designation**	Rural	Rural	Rural	Rural	Urban	Urban	Rural	Urban	Urban	Rural
**Staffed Beds**	94.7	224.1	56.6	49.0	58.6	53.4	115.9	290.3	28.0	411.4
**Inpatient Revenue (Millon)**	197.14	688.61	150.82	12.68	64.72	88.33	669.52	2516.52	25.24	1398.71
**Outpatient Revenue (Millon)**	780.05	1480.14	625.21	30.62	1477.44	134.31	661.67	2054.37	124.32	2214.55
**VBP Quality Score**	33.1	25.2	31.8	22.4	44.0	43.2	42.0	23.0	55.0	23.2
**Readmission Rate**	15.4%	15.0%	14.7%	16.6%	14.8%	15.2%	14.9%	15.7%	14.5%	15.5%
**Contract Labor (Millon)**	3.97	10.15	3.60	0.10	1.65	0.15	3.17	5.45	0.41	100.71
**Outpatient Visits**	271,722	162,880	68,305	19,190	227,795	51,008	54,428	866,200	17,518	911,290
**Total Surgeries**	3,992	13,786	6,802	388	3,796	1,476	6,048	16,799	1,355	18,683
**IT Operating Expense (Millon)**	7.51	10.22	3.96	0.64	5.86	1.74	1.50	21.70	1.28	57.22

Note: Hospital have been renamed HospE1, HospE2, etc. to deidentify the results.

Table S2. Top 10 Least Efficient Hospital-Year Observations

Variable	HospI1	HospI2	HospI3	HospI4	HospI5	HospI6	HospI7	HospI8	HospI9	HospI10
**DEA Efficiency Score**	0.14	0.15	0.17	0.19	0.19	0.19	0.21	0.21	0.21	0.21
**Rural / Urban Designation**	Urban	Urban	Urban	Urban	Urban	Urban	Urban	Urban	Urban	Urban
**Staffed Beds**	301.7	231.1	231.7	139.6	114.0	170.0	245.4	130.1	228.0	102.0
**Inpatient Revenue (Millon)**	232.71	333.47	449.29	127.74	135.23	202.47	343.47	150.61	319.81	113.17
**Outpatient Revenue (Millon)**	270.68	273.71	274.65	230.97	162.32	149.49	527.26	215.32	319.30	192.72
**VBP Quality Score**	24.6	30.3	27.4	28.4	62.5	33.2	26.5	24.8	47.7	43.5
**Readmission Rate**	16.4%	16.6%	16.8%	14.9%	15.0%	14.1%	15.4%	16.0%	15.2%	15.7%
**Contract Labor (Millon)**	0.39	0.35	1.37	0.18	37.88	7.94	107.06	2.24	10.59	5.30
**Outpatient Visits**	85,709	86,284	92,096	116,963	139,079	35,500	232,646	33,229	106,149	97,890
**Total Surgeries**	4,722	7,065	5,283	7,598	1,971	4,267	4,260	3,516	10,723	5,047
**IT Operating Expense (Millon)**	8.13M	8.68M	14.15	4.58	7.07	5.81	19.31	3.23	10.84	4.31

Note: Hospital have been renamed HospI1, HospI2, etc. to deidentify the results.
